# The pathophysiological mechanisms underlying diabetic retinopathy

**DOI:** 10.3389/fcell.2022.963615

**Published:** 2022-08-30

**Authors:** Lindan Wei, Xin Sun, Chenxi Fan, Rongli Li, Shuanglong Zhou, Hongsong Yu

**Affiliations:** ^1^ Special Key Laboratory of Ocular Diseases of Guizhou Province, Department of Immunology, Zunyi Medical University, Zunyi, China; ^2^ Special Key Laboratory of Gene Detection and Therapy of Guizhou Province, School of Basic Medical Sciences, Zunyi Medical University, Zunyi, China

**Keywords:** diabetic retinopathy, oxidative stress, inflammation, neovascularization, neurodegeneration, neurovascular unit, gut microbiota

## Abstract

Diabetic retinopathy (DR) is the most common complication of diabetes mellitus (DM), which can lead to visual impairment and even blindness in severe cases. DR is generally considered to be a microvascular disease but its pathogenesis is still unclear. A large body of evidence shows that the development of DR is not determined by a single factor but rather by multiple related mechanisms that lead to different degrees of retinal damage in DR patients. Therefore, this article briefly reviews the pathophysiological changes in DR, and discusses the occurrence and development of DR resulting from different factors such as oxidative stress, inflammation, neovascularization, neurodegeneration, the neurovascular unit, and gut microbiota, to provide a theoretical reference for the development of new DR treatment strategies.

## Introduction

**FIGURE 1:**
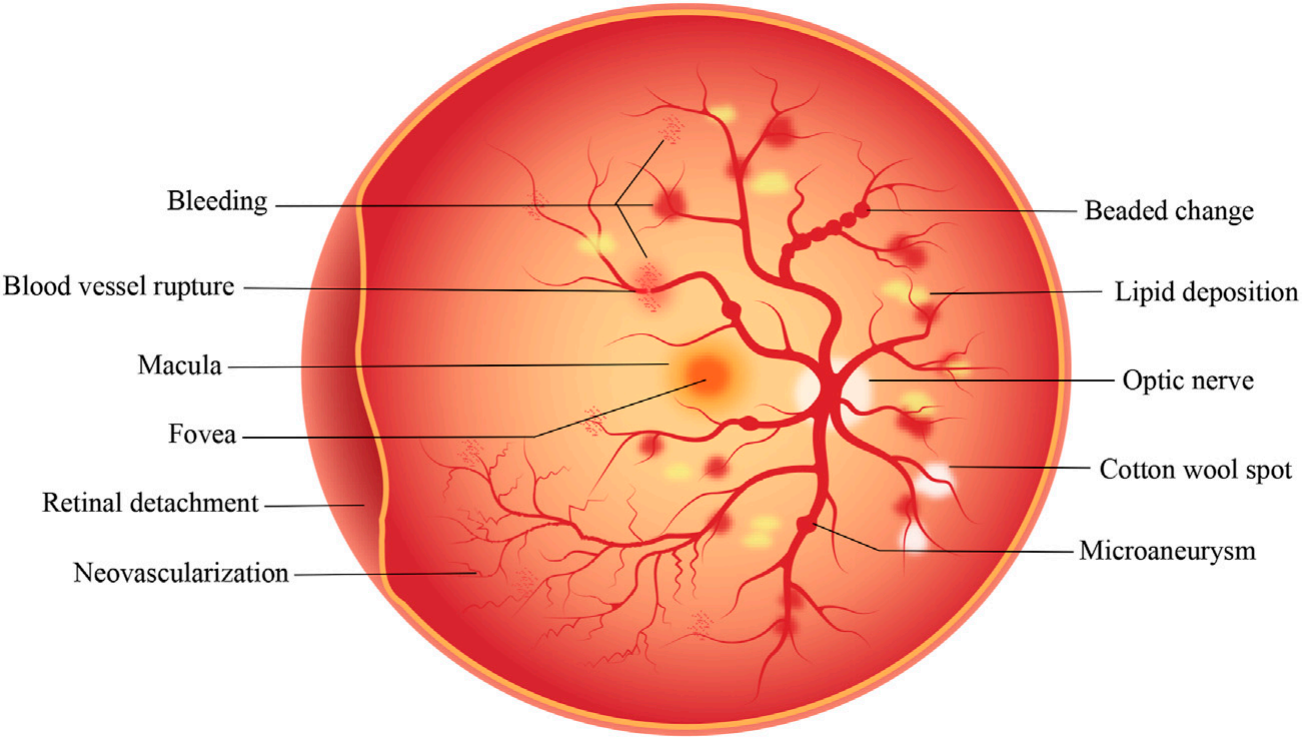
Pathological changes that occur on the retina in DR. The development of DR is a gradual process. In the early stage of NPDR, microaneurysm, cotton wool spots and minor bleeding may occur. In the later stage of PDR, serious conditions such as vascular rupture, severe bleeding, retinal detachment and neovascularization may occur.

Diabetic retinopathy (DR) is the most common complication of diabetes mellitus (DM) (Whitehead et al., 2018). It is an irreversible blinding eye disease that imposes a severe burden on patients, healthcare systems, and society throughout the world (Simó et al., 2020; Antonetti et al., 2021). The International Diabetes Federation has estimated that the number of DM patients worldwide will increase from 463 million in 2019 to 700 million by 2045 (Saeedi et al., 2019). Teo et al. estimated that, as 22.27% of DM patients develop DR, the global prevalence of DR will increase from 103.12 million in 2020 to 160.5 million by 2045 (Teo et al., 2021). DR can be classified into non-proliferative diabetic retinopathy (NPDR) and proliferative diabetic retinopathy (PDR) according to the disease process. NPDR, known as early DR, is characterized by clinical manifestations such as retinal microaneurysms, cotton wool spots, hemorrhages, and exudates, of which the patient may not be aware. NPDR progresses to a more severe disease over time. PDR, also known as late DR, is typically characterized by the formation of new blood vessels that are very fragile and can thus rupture and bleed profusely (Solomon et al., 2017). The proliferation and migration of retinal pigment epithelial (RPE) cells, together with the secretion of extracellular matrix components by the cells, contribute to the distinctive fibrotic membranes formed at the vitreoretinal interface in PDR. Vascular endothelial growth factor (VEGF) and connective tissue growth factor (CCN2/CTGF) contribute to blindness through their promotion of neovascularization and subsequent fibrosis. It has been reported that CCN2 and the CCN2/VEGF ratio are the most powerful predictors of the extent of fibrosis (Kuiper et al., 2008). As DR progresses, retinal detachment, hemorrhage, and irreversible visual impairment occur. Figure 1 shows the pathological changes in the retina that occur in DR. Despite significant progress in the recognition and treatment of DR, its pathogenesis remains understudied, and the prevalence of DM, as well as DR, is still increasing. Therefore, it is crucial to study the specific pathogenesis of DR to develop new therapeutic strategies.

### DR and oxidative stress

DR is associated with a variety of stress mechanisms, among which the role of oxidative stress is crucial. In the early stage of DR, hyperglycemia leads to the formation of reactive oxygen species (ROS), composed of superoxide ions, hydrogen peroxide, and hydroxyl radicals. ROS are by-products of cellular metabolism, mainly derived from macrophages, mitochondrial electron transport chain, and mitochondrial lipid peroxidation. There are, however, antioxidants that actively scavenge ROS. In situations of excessive formation and reduced clearance of ROS, ROS accumulation in vascular endothelial cells leads to adverse effects mediated by activation of the polyol, protein kinase C (PKC), and hexosamine pathways, as well as the presence of increased amounts of advanced glycation end products (AGEs) and increased expression of AGE receptors together with their activating ligands (Kowluru et al., 2015; Kang and Yang, 2020). This leads to oxidative stress *in vivo*, which can result in pyroptosis, apoptosis, and autophagy (Lin and Kuang, 2014), promoting inflammation, vascular degeneration, neurodegeneration, and neovascularization (Rodríguez et al., 2019).

In addition to the well-known mechanisms described above, other mechanisms, including mitochondrial dysfunction (Ravikumar et al., 2004), decreased Nrf2 activity (Boland et al., 2008), epigenetic modification (Kowluru, 2020), and the activation or inhibition of multiple signaling pathways, have also been implicated in oxidative stress. The mitochondria in cells function as energy manufacturing factories. During adenosine triphosphate (ATP) synthesis and oxidative phosphorylation, the electron transport chain generates ROS (Dan Dunn et al., 2015), and the increase in ROS further damages mitochondrial DNA, resulting in abnormal mitochondrial function and reduced retinal function, ultimately leading to retinal damage produced by oxidative stress (Garcia Soriano et al., 2001; Madsen-Bouterse et al., 2010). It can be seen that the relationship between the mitochondria and ROS is both iterative and bidirectional. Nuclear factor E2-related factor 2 (Nrf2) is a redox-sensitive transcription factor. Wang et al. reported that silencing of Nrf2 led to increased ROS production and apoptosis in rat retinal pericytes (RRPs), a decreased B-cell lymphoma-2 (Bcl-2) to Bcl-2-associated X (BAX) ratio, and increased expression of heme oxygenase, nicotinamide adenine dinucleotide phosphate (NADP) quinone redox reductase, superoxide dismutase 2 (SOD2), catalase, and glutamate-cysteine-ligase catalytic and modification subunits. The oxidative stress sensor DJ-1 is encoded by the *PARK7* gene and belongs to the C56 peptidase family. Overexpression of DJ-1 was found to induce Nrf2 expression leading to the controversial result (Wang et al., 2020). Zhou et al. found that forkhead box O6 (*FOXO6*) was overexpressed in vitreous samples from DR patients and a high-glucose (HG)-induced human RPE cell line (ARPE-19) and that *FOXO6* knockdown activated the Akt/Nrf2 signaling pathway (Zhou et al., 2019). These findings suggest that reductions in Nrf2 activity lead to increased oxidative stress and that Nrf2 activation may be a key strategy for the prevention of oxidative stress. However, we consider that H-RPE primary cells should also be included in investigations with these cellular models, as H-RPE primary cells maintain many important signs and functions *in vivo*. Both ARPE19 cells and H-RPE primary cells have been used as cell culture models, with H-RPE primary cells accepted as an effective primary cell culture model in recent years (Hwang et al., 2019; Udsen et al., 2022; Yang et al., 2022).

Epigenetic modifications, such as DNA methylation, histone methylation, histone acetylation, dysregulated miRNA, and lncRNA expression, mediate changes in the expression of genes associated with key regulatory pathways. Epigenetic modifications have been reported in a variety of cells in DR, together with observations of metabolic memory (Kato and Natarajan, 2019; Gao et al., 2021). Kowluru et al. demonstrated that methylation of Lysine9 on histone H3 (H3K9me3) on the Ras-related C3 botulinum toxin substrate 1 (Rac1) promoter activated Rac1 transcription, and regulation of Suv39H1-H3K9 trimethylation prevents further epigenetic modifications as well as the occurrence and development of DR (Kowluru et al., 2021).

It is well-known that high glucose levels increase the expression of lncRNA MALAT1 by increasing the binding of the Sp1 transcription factor to its promoter region, and a recent study identified a role of lncRNA MALAT1 in the regulation of the Keap1-Nrf2-antioxidant defense system in DR (Radhakrishnan and Kowluru, 2021). These authors found that retinal microvessels from STZ-induced diabetic mice and DR patients showed similar increases in lncRNA MALAT1, as well as confirming its interaction with Keap1 and decreased expression of Nrf2-mediated antioxidant defense genes. It can thus be seen that lncRNA MALAT1 can regulate the antioxidant defense system in DR through Keap1-Nrf2, suggesting that inhibition of the antioxidant defense system contributes significantly to DR pathogenesis, as has also been proposed by other authors (Kang and Yang, 2020; Saddala et al., 2020). Oxidative stress can also exert its effects through multiple signaling pathways, including the AMPK/TSC1/TSC2/RHEB pathway. AMPK can regulate the glucose metabolism pathway and can also inhibit the mTOR signaling pathway, ultimately leading to the induction of autophagy (Zhao et al., 2019).

The significance of oxidative stress is thus self-evident. It is the central link in the pathophysiology of DR and has a connecting role. Several studies support that oxidative stress should be considered a “unifying mechanism” in DR pathogenesis (Brownlee, 2005; Li et al., 2017). This suggests that antioxidant therapy would be a useful early adjuvant treatment for DR.

### DR and inflammation

In addition to the oxidative stress seen in early DR, chronic low-titer inflammation of the retina may also occur. As early as 1948, DR was known as “diabetic retinitis” (RODRIGUEZ and ROOT, 1948), and the role of pro-inflammatory mechanisms in DR has received considerable attention. Early-stage DR is characterized by various inflammation-associated features, including increased vascular permeability, increased retinal blood flow, neutrophil infiltration, macrophage infiltration, glial activation, complement activation, and tissue edema (Forrester et al., 2020). Several clinical studies have shown that the use of anti-inflammatory drugs can reduce both vascular permeability and VEGF expression, preventing cell death (Semeraro et al., 2019). This suggests that anti-inflammatory treatment is beneficial for DR.

DR-associated inflammation has been found to be mediated by factors such as leukocyte arrest, inflammation-related cytokines (such as interleukin-1β), miRNA regulation, and the activation or inhibition of signaling pathways. Studies using mice deficient in the genes encoding the cluster of differentiation 18 (CD18) and intercellular adhesion molecule 1 (ICAM-1) showed that leukocyte stasis increased within a few days after the onset of diabetes and was associated with increased expression of CD18 and ICAM-1 in the retina (Joussen et al., 2004). Despite its early onset and short duration, leukocyte stasis is considered to be a central event in inflammation (Joussen et al., 2004). However, Van et al. considered that leukocyte stasis is merely an incidental phenomenon occurring during DR (van der Wijk et al., 2017). Is leukocyte stasis associated with DR? Although this issue remains controversial, the centrality of inflammation in DR is clear. Feng et al. investigated the overexpression of high mobility group box-1 (HMGB1) resulting from exposure to high glucose, and found that overexpression of HMGB1 led to lysosomal membrane permeability (LMP) by upregulating the expression of lysosomal enzyme B; conversely, inhibition of HMGB1 expression could rescue LMP, restore autophagy-mediated degradation, reduce the expression of inflammatory factors and VEGF, and have a protective effect on apoptosis in RPE cells in the early stages of DR (Feng et al., 2022). The involvement of multiple inflammatory-associated factors has been reported in DR, including ICAM-1, CD18, IL-1β, IL-3, IL-6, IL-8, tumor necrosis factor-α (TNF-α), monocyte chemotactic protein 1 (MCP-1), macrophage inflammatory protein 1 (MIP-1), CCL3, CCL15, and CCL21. Interestingly, some studies have found that CCL3 may be involved in the development of early DR, and CCL15 and CCL21 may be closely related to the formation of fibrovascular membranes and the progression to end-stage DR (Zeng et al., 2019), suggesting that the inflammatory response runs through DR development. Although inflammatory factors may have some protective effects in early-stage DR, the persistence of inflammation can cause cytotoxic damage. For example, the barrier effect of vascular wall pericytes caused by inflammation disappears (Ogura et al., 2017). Damage to the blood-retinal barrier accelerates the progression of DR. IL-1β is an essential inflammatory mediator derived from endothelial cells and microglia. IL-1β can be activated by nuclear factor-κB (NF-κB) to induce pericyte apoptosis, and it can also reduce the numbers of tight junction proteins in endothelial cells, ultimately enhancing endothelial cell permeability (Yun, 2021).

The regulatory mechanisms of miRNAs in DR have recently become a research hotspot. Several miRNAs have been found to influence biological processes in the retina by regulating gene expression (Bao and Cao, 2019). MiRNAs are found in adipose tissue and obesity is closely associated with chronic low-degree inflammation. Furthermore, miRNAs can directly influence the expression of inflammation-associated proteins and can also alleviate the development of DR by agonists, targeted inhibition of transcription, and other methods (Yang et al., 2020). MiRNAs are thus worthy of in-depth study and may be effective targets or biomarkers of DR treatment. Several inflammation-related signaling pathways are also activated in early-stage DR, such as the JAK-2/STAT3, PI3K/AKT, and MAPK signaling pathways; these pathways are also linked by common enzymatic reactions that are similar to signaling networks promoting the development of DR. The most-studied pathway is the PI3K/AKT signaling pathway, downstream components of which are associated with VEGF signaling, with the combined cascade of these signaling pathways ultimately driving VEGF expression and leading to vascular degeneration.

The most commonly used anti-inflammatory drugs are corticosteroids and non-steroidal anti-inflammatory drugs. The advantages of corticosteroids are the need for less frequent intraocular injections and their lower cost. However, adverse effects are common, and the overall efficacy of corticosteroids for treating PDR remains uncertain, and corticosteroids are thus not recommended for patients with PDR. IL-6 is one of the most important pro-inflammatory cytokines present in the vitreous of DR patients, Inhibition of IL-6 trans-signaling can significantly reduce oxidative stress in retinal endothelial cells and prevent retinal oxidative damage. Antibodies against IL-6 and IL-6 receptors have been developed and are currently undergoing clinical trials (Robinson et al., 2020).

### DR and neovascularization

Neovascularization develops gradually over time, starting with the destruction of the basement membrane surrounding the microvascular endothelial cells, thus allowing movement of the endothelial cells in the direction of chemokines. These migrating endothelial cells elongate, divide and eventually form tubular structures that gradually fuse over time, leading to the formation of new mature capillaries. It is well known that neovascularization plays an important role in DR pathogenesis. A summary of this pathological process is as follows: elevated blood glucose → retinal ischemia and hypoxia → capillary endothelial cell damage → capillary endothelial cell repair → capillary basement membrane hyperplasia and thickening → capillary lumen narrowing → aggravation of retinal ischemia and hypoxia → aggravation of capillary endothelial cell damage → microaneurysm formation → capillary occlusion → complete tissue hypoxia → capillary necrosis → neovascularization (Heitzig et al., 2017; Lechner et al., 2017; Scanlon, 2017). In terms of the timeline of DR development, it is apparent that early DR (NPDR) does not have any clinical symptoms, although obvious clinical discomfort may appear in late DR (PDR). In the final stages of the disease, the retina is characterized by neovascularization or hemorrhage.

Pericyte apoptosis, activation and inhibition of signaling pathways by multiple key factors, and increased VEGF all play crucial roles in neovascularization. Pericytes are important components of retinal blood vessels and their apoptosis can lead to increased vascular permeability, vascular occlusion, and microaneurysm formation. Mizutani et al. found that pericyte apoptosis in the retinas of DM patients was significantly accelerated compared with the control group (Mizutani et al., 1996). Of the various degenerative changes seen in DR microvessels, pericyte apoptosis and the presence of “pericyte ghosts” are the key histopathological hallmarks of DR. Cheng et al. reported that prostaglandin F2α (PGF2α) can reduce pericyte apoptosis by inhibiting the PI3K/Akt/GSK3β/β-catenin signaling pathway (Cheng et al., 2021). NEAT1 is a long non-coding RNA discovered in recent years. NEAT1 silencing can inhibit oxidative stress in human retinal endothelial cells, inhibit pro-inflammatory signaling, and also reduce the expression of VEGF (Shao et al., 2020). Gong et al. suggested that Roundabout 4 (ROBO4) was involved in neovascularization and that its expression varies with DR severity, and they found that targeting HIF-1α/SP1-dependent ROBO4 expression by miR-125b-5p/miR-146a-5p could delay DR progression (Gong et al., 2019). There are still many signaling pathway regulatory mechanisms to be explored, and the in-depth study of signaling pathways has become a fundamental way to explain disease mechanisms. The angiopoietin (Ang)/Tie2 signaling axis is a key regulator of neovascularization. The combination of Ang-2 and Tie2 destroys the stability of local blood vessels, causes vascular leakage, promotes vascular endothelial cell migration, and ultimately leads to pathological neovascularization. Ang plays an important role in regulating vascular remodeling and maturation in the later stages of neovascularization. It has been proposed that inhibition of Ang-2 expression through regulation of the Ang/Tie2 signaling axis may be a potential therapeutic strategy for DR patients (Brkovic et al., 2007; Campochiaro and Peters, 2016). Currently, in addition to anti-VEGF drugs, several anti-angiogenic drugs are in clinical trials, including squalamine, AKB-9778 (Tie2 activator), Nesvacumab (anti-Ang-2), and RO6867461 (bispecific antibody: anti-Ang-2 + anti-VEGF). The discovery of these drugs is based on in-depth research into the mechanism of DR (Wang and Lo, 2018).

The growth factors closely related to DR include VEGF, erythropoietin (EPO), insulin-like growth factor (IGF), transforming growth factor-β (TGF-β), basic fibroblast growth factor (BFGF), CCN2/CTGF, hematopoietic growth factor (HGF), platelet-derived growth factor (PDGF), and epidermal growth factor (EGF). Although these growth factors have all been shown to be associated with DR, it was recently found that EPO can maintain the internal blood-retinal barrier (IBRB) by activating Src/Akt/cofilin signaling in microglia and inhibiting microglial phagocytosis. Although these data support the potential of EPO in the treatment of DR, VEGF still remains the research hotspot (Xie et al., 2021). VEGF is an angiogenic factor and it is the strongest selective mitogenic factor for endothelial cells. VEGF-A is the most important member of the VEGF family, and binds to the key receptor VEGFR2, stimulating the proliferation, migration, survival, and increased permeability of vascular endothelial cells, ultimately leading to neovascularization (Ferrão et al., 2019). Although VEGF is a causative factor for PDR, it is also required for normal vascular growth and plays a crucial role in maintaining endothelial cell integrity. Mima et al. found that while inflammation and oxidative stress can induce VEGF expression, they also suggested that, in addition to inflammation and oxidative stress, factors such as persistent hyperglycemia may induce VEGF expression (Mima et al., 2012). Excessive VEGF expression can disrupt the intracellular barrier, increase leakage of the choroid plexus endothelium, cause edema, and activate inflammatory signaling pathways (Kinoshita et al., 2016). Recently, Hachana et al. found that the anti-VEGF and B1 receptor antagonist (R-954) reduced the levels of B1 and B2 receptors, TNF-α, and ICAM-1 in choroidal neovascularization, and also downregulated the expression of VEGF-A, VEGF-R2, HIF-1α, CCL2, and VCAM-1 (Hachana et al., 2020). These findings indicate both the complexity of DR pathogenesis and the close relationships between the responsible mechanisms. Clinically, the most commonly used drugs for treating DR are anti-VEGF substances that can protect the function of the vascular wall, reduce retinal microvascular leakage, alleviate local ischemia and hypoxia, and inhibit neovascularization (Bressler et al., 2017). These anti-VEGF drugs include pegaptanib, ranibizumab, aflibercept, and bevacizumab. Intravitreal ranibizumab has been shown to benefit patients more than laser therapy (Massin et al., 2010) while aflibercept was found to be superior to ranibizumab and bevacizumab in improving vision in patients with moderate or severe vision loss (Wells et al., 2015). The method of injecting anti-VEGF drugs directly into the eyes of patients has been widely used in the treatment of DR. Although this invasive approach cannot completely cure DR, anti-VEGF drugs are still the first choice of first-line treatment.

### DR and neurodegeneration

Although the clinical classification of DR is based on the degree of neovascularization in patients, several studies have reported the presence of neuronal and glial changes in the retina before neovascularization (Carpineto et al., 2016). From another perspective, DR can also be regarded as a neurodegenerative disease. Neurodegeneration is often associated with mechanisms such as oxidative stress, impaired antioxidant defense mechanisms, imbalance of neuroprotective factors, glutamate excitotoxicity, mitochondrial dysfunction, activation of the renin-angiotensin system, and phosphorylation of the microtubule-associated protein tau (Masser et al., 2017; Sinclair and Schwartz, 2019; Pillar et al., 2020). The roles of oxidative stress and neovascularization in DR have also been extensively studied. Studies have found that in the retinas of diabetic mice, inhibition of ROS production can effectively block DR development and the caspase-3-mediated apoptosis of retinal neurons. It is apparent that these roles do not exist independently but are interrelated (Sasaki et al., 2010). Studies have shown that compared with non-diabetic patients, the retinas of diabetic patients have reduced levels of a variety of neuroprotective factors, such as pigment epithelium-derived growth factor (PEDF), somatostatin (SST), and interstitial retinol-binding protein (IRBP). Reduced expression of IRBP is observed in the very early stages of DR and has also been associated with retinal neurodegeneration. In addition to the down-regulation of neuroprotective factors and up-regulation of neurotrophic and survival factors, VEGF and EPO have been found to be overexpressed in patients with early DR. As mentioned earlier, neurodegeneration is thought to occur before vascular degeneration, suggesting the possibility that factors that have been linked to early-stage DR may be more closely associated with neurodegeneration than with blood vessels. This deserves attention. In a recent American Diabetes Association statement, DR was defined as a highly tissue-specific neurovascular complication, and it can be observed that the analysis of nerves or blood vessels alone is becoming less and less meaningful. Thus, blood vessels and nerves are inextricably linked. We should thus not define DR from a single perspective but should consider the interrelationships between multiple factors for a full understanding of the complexity of this disease (Oshitari, 2021).

When we realize that neurodegeneration is an early event in DR, therapeutic drugs based on neuroprotection become meaningful. Such drugs include somatostatin, nerve growth factor (NGF) and brain-derived neurotrophic factor (BDNF) (Simó and Hernández, 2014). In addition to confining the therapeutic targets to neurons, we should aim to restore the functions of damaged Müller cells and microglia. We should no longer limit DR treatment to one method only but should instead adopt a comprehensive treatment strategy, such as focusing on preventing the leakage of blood vessels, inhibiting the formation of new blood vessels, rebuilding the blood supply of the retina, and strengthening protection of the nerves, representing the ultimate goal for treating DR. It is important to realize that DR is a disease characterized by cross-links and interrelationships between nerves and blood vessels, and retinal dysfunction is the product of a complex array involving multiple inter-dependent mechanisms.

### DR and NVU

The concept of the neurovascular unit (NVU) was first formalized at the 2001 National Institute of Neurological Disorders and Stroke’s Stroke Progress Review Group meeting and has since been applied to the retina (Iadecola, 2017). The NVU includes neurons, glia, immune cells, and vascular cells; however, both the functional coupling and interdependence among these cells are important for maintaining hemostatic function and regulating neuronal activity (Liu et al., 2019). All the constituent cells of the NVU are integrated to maintain the integrity of the inner blood-retinal barrier and to dynamically regulate local blood flow (Simó and Hernández, 2014). Disruption of the NVU may lead to the structural or functional impairment of both microvessels and neurons. NVU lesions can be caused by a variety of factors, such as neuronal loss, glial cell loss, and changes in the vasculature (Xia et al., 2021). There are several very interesting points concerning our knowledge of the NVU, namely, the influence of changes in associated factors and signal transmission, and the influence of Müller cells. Active neurotrophic factors are essential for retinal homeostasis, and when these factors are up-regulated, they produce strong angiogenic results in addition to their neuroprotective effects (Simó et al., 2014). Among them, VEGF, IGF-1, EPO, and secretogranin III (SCGIII) are the most important. Hernandez et al. showed that the pro-angiogenic effect of VEGF was weak during early-stage DR where the expression and release of VEGF functioned only to protect retinal neurons (Hernández et al., 2016). Hombrebueno et al. found that low levels of amacrine and ganglion cell death and albumin leakage occurred after continuous injection of anti-VEGF drugs in Akita mice (Hombrebueno et al., 2015). These results show that reducing VEGF levels can aggravate retinal neurodegeneration. In contrast, Rojo Arias et al. found that applying aflibercept (VEGF-Trap) to OIR mice not only alleviated retinal microvascular aberrations but also significantly improved the neural function of the retina (Rojo Arias et al., 2020). The reason for the discrepancies in these findings may be due to different drug dosages, different methods of drug use, and the different time nodes of onset. However, the findings indicate that these neuroprotective effects require further study. IGF-1 is considered to be a pleiotropic factor, mainly produced by endothelial cells, pericytes, and RPE cells. It acts as a survival factor for retinal microvascular endothelial cells and nerve cells and can play a key role in retinal immune regulation (García-Ramírez et al., 2008; Arroba et al., 2018). EPO overexpression is an early event in the retina of diabetic patients, and EPO is a potential neuroprotective factor that can mobilize endothelial progenitor cells to damaged retinal sites and is involved in the reconstruction of tissue injury (Chen et al., 2008). Similar to VEGF, SCGIII is also a pro-angiogenic factor similar to VEGF but, while VEGF is expressed in both normal and abnormal blood vessels, leading to potential drawbacks associated with anti-VEGF therapy, the expression of SCGIII is very specific. Li et al. found that the specific expression of SCGIII in retinal blood vessels of diabetic patients was as high as 97.69% with no specific expression in normal peripheral capillaries, and that expression of the SCGIII receptor was significantly increased in diabetic neonatal retinal vascular endothelial cells (Li et al., 2018). LeBlanc et al. observed that anti-SCGIII agents could prevent retinal neovascularization in mice with hypoxia-induced retinopathy, and suggested that SCGIII had great potential as a novel target for DR therapy (LeBlanc et al., 2017).

The NVU includes Müller cells, astrocytes, ganglion cells, amacrine cells, horizontal cells, bipolar cells, endothelial cells, pericytes, microglia, macrophages, and basement membrane. Recently, Alarcon-Martinez found that interpericyte tunneling nanotubes (IP-TNTs) can mediate signal transmission between pericytes, and that the NVU can also maintain homeostasis by regulating blood flow, vascular density, and vascular permeability (Alarcon-Martinez et al., 2020). In the NVU, neurons receive nutrients and eliminate metabolic wastes by regulating vascular activity (Nian et al., 2021), and glial cells play a synergistic role in blood vessels to eliminate metabolites, transmit neurotransmitters, maintain homeostasis, and transduce signals (Filosa et al., 2016). A characteristic change seen in DR is the increased expression of glial fiber acidic protein in the Müller cells. Furthermore, while activation of Müller cells is not the cause of neurodegeneration, it promotes neuroprotection through the activation of extracellular signal-regulated kinase 1/2 (ERK1/2) (Matteucci et al., 2014). Müller cells are closely connected with both retinal blood vessels and neurons, and Müller cells play important roles in glutamate metabolism, extracellular ion homeostasis, and neuronal function. Both Müller cells and ganglion cells secrete BDNF, and it has been found that down-regulation of BDNF expression in the diabetic retina can protect retinal neurons through the TrkB/ERK/MAPK signaling pathway (Liu et al., 2013; Le et al., 2021). These findings suggest that appropriate supplementation of neurotrophic factors could improve DR.

As hyperglycemia can cause changes in all the cells within the retinal NVU, the NVU could be used as one of the early therapeutic targets for DR (Gardner and Davila, 2017; Kugler et al., 2021; Simó et al., 2021). The same strategies for treating neovascularization and neurodegeneration apply to the NVU. In general, rather than studying the roles of the neurovascular cells only, we would recommend studying the NVU as a whole, especially as an early warning of DR, which would be helpful for the early prevention and early treatment of DR.

### DR and gut microbiota

Evidence suggests that the composition, modification, and disturbance of the gut microbiota can affect important physiological processes *in vivo* and that the gut microbiota may contribute to diseases including chronic inflammation, immune system imbalances, type 2 diabetes, chronic kidney disease, and rheumatoid arthritis (Scirocco et al., 2016; Schoeler and Caesar, 2019; Yang C Y et al., 2021). Similarly, the close relationship between the gut microbiota and uveitis and glaucoma, as well as age-related macular degeneration, illustrates the significance of the gut microbiota to disease susceptibility and development (Jiao et al., 2021). The gut microbiota forms a crucial part of the body and plays a powerful role in the maintenance of normal metabolism and immune regulation. Changes in environmental, immune, genetic, or host factors can disrupt the balance of the gut microbiota. Such disturbance in association with hyperglycemia can lead to disruption of the gut barrier, resulting in damage to multiple organs. Dysregulation of the gut microbiota by hyperglycemia in patients with type 2 diabetes leads to bacterial translocation and intestinal endotoxin accumulation, resulting in intestinal inflammation (Yang G et al., 2021). Lipopolysaccharide (LPS) derived from *Bacteroides* and Desulfobacteria in the gut is a major substance associated with microbial translocation during chronic inflammation, and LPS can accumulate in the gut and enter the bloodstream, causing metabolic endotoxemia. Vagaja et al. found that systemic exposure to LPS in Ins2 Akita mice may accelerate retinal capillary endothelial cell damage, suggesting that endotoxin-mediated retinal inflammation may affect the DR phenotype (Vagaja et al., 2013). Gut microbes are not only associated with inflammation but also with neurodegeneration. High glucose levels in the body can induce the expression of Toll-like receptor (TLR-4) in retinal ganglion cells, resulting in the expression of TNF-α, IL-8, and NF-κB and promoting activation of microglial glycosylase, as well as increased vascular permeability, ultimately resulting in neuronal damage (Campo et al., 2010). The composition of the gut microbiota can affect the levels of LPS, short-chain fatty acids (SCFA), bile acids, trimethylamine (TMA), and tryptophan (TRP) in the blood circulation. DM complications have been reported to be associated with the gut microbiota, leading to insulin resistance, chronic inflammation, and imbalances in immune regulation (Huang et al., 2021). It has been found that tryptophan, serotonin, and canine uric acid are closely related, and they can reflect the functioning of the “intestinal-brain axis” (Yano et al., 2015).

It is well known that there is a close relationship between retinal and brain microvessels. Several recent studies have proposed the concept of a “gut-retinal axis” and have investigated the relationships between the gut microbiota, DM, and DR. Rowan et al. found that the gut microbiota and its metabolites (tryptophan, serotonin, and maluric acid) can act within the “gut-retinal axis” (Rowan et al., 2017). Huang et al. investigated the changes in the composition of the gut microbiota in DM and DR and constructed a microbiota-based model that could effectively distinguish between DR and DM patients, as well as identify potential therapeutic targets for DR (Huang et al., 2021). Andriessen et al. demonstrated that high-fat diet-induced obesity leads to gut microbiota dysbiosis, which was found to drive retinal inflammation and pathological neovascularization in a mouse model of laser photocoagulation-induced choroidal neovascularization (CNV) (Andriessen et al., 2016). This series of studies have suggested potential mechanisms through which the gut microbiota affects the pathophysiology of ocular diseases. Therefore, we can boldly speculate that there is a strong association between both the gut microbiota and its metabolites, suggesting potential as future biomarkers for the clinical diagnosis and treatment of DR.

All in all, the gut microbiota is now considered to represent an additional human organ that has co-evolved with the host. Growing evidence suggests the crucial role played by the microbiota in the development of T2DM. Dysregulation of the gut microbiota leads to persistent inflammation and immunosenescence, both of which may be involved in the pathogenesis of DR. Beli et al. found that INT-767 prevented DR in a mouse model of diabetes. Notably, INT-767 is a potent and selective agonist and modulator of the farnesoid X receptor (FXR) and bile acid G protein-coupled membrane receptor 5 (TGR5) (Beli et al., 2018). Tauroursodeoxycholate (TUDCA) is an endoplasmic reticulum stress inhibitor and a neuroprotective secondary bile acid. TUDCA can affect glucose and lipid metabolism by regulating FXR and TGR5. Overall, changes in the gut microbiota lead to the production of TUDCA, which promotes TGR5 activation in retinal neurons to prevent DR. At present, there is insufficient research on the “gut-retinal axis” and there has been no dynamic monitoring of the gut microbiota. It is thus necessary to conduct longer-term and sustained research observations, and to further study the role of gut microbiota in DR from the cellular and molecular levels through multi-omics approaches, which will help us understand the underlying mechanism of the “gut-retinal axis”. At present, there is no specific drug for the treatment of DR, and it may be possible to use the gut microbiota as an interventional measure for DR by adjusting the types, quantity, and microenvironments of the gut microbiota, and to predict the risk of DR.

## Conclusion

**FIGURE 2:**
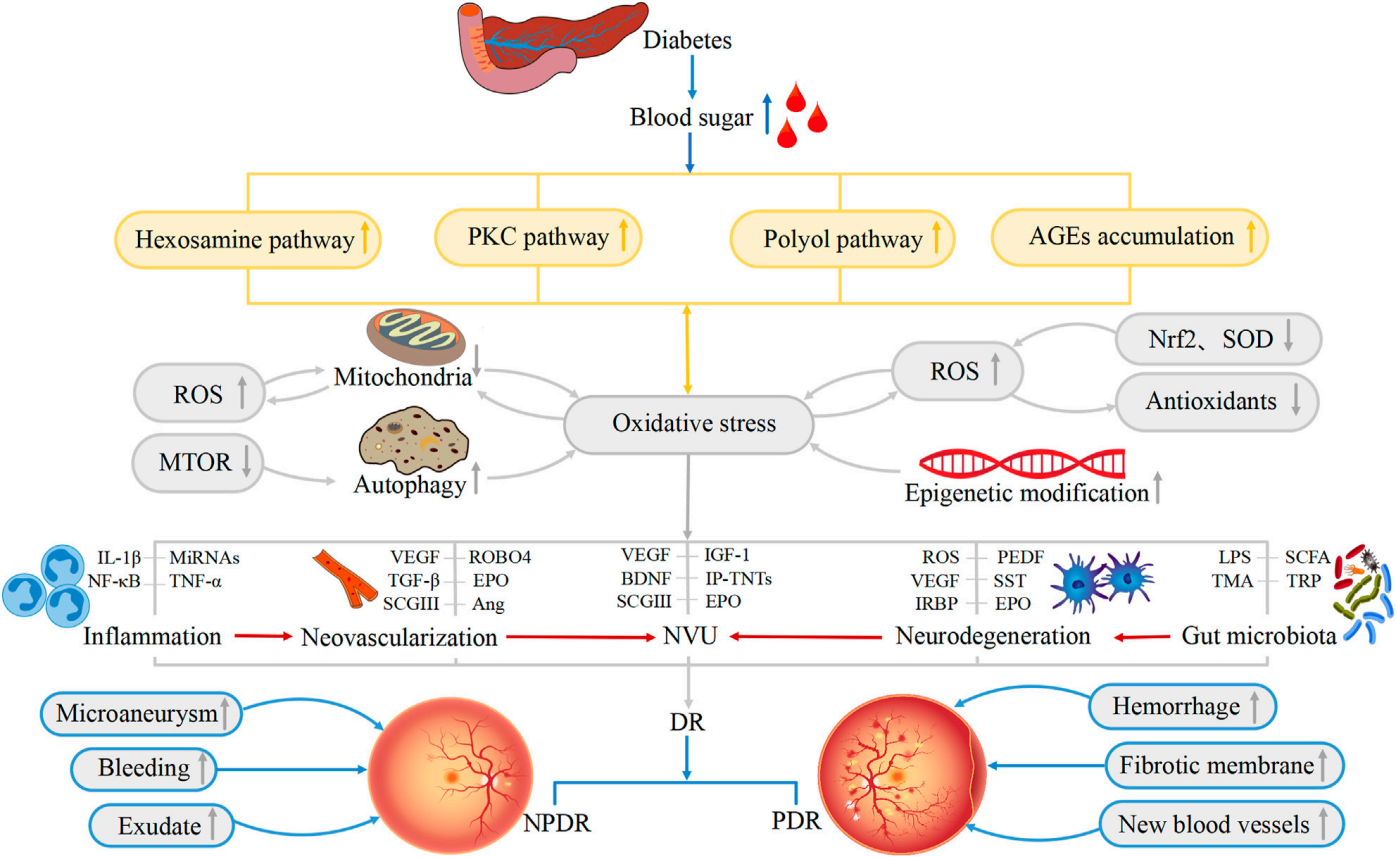
Physiological changes associated with the development of DR. Hexosamine pathway, polyol pathway, PKC pathway and AGEs accumulation lead to the occurrence of oxidative stress. Oxidative stress, inflammation, neovascularization, neurodegeneration, NVU, and gut microbiota are all involved in the pathogenesis of DR. ROS, Nrf2, SOD, IL-1β, VEGF, CSGIII, LPS and other substances also directly or indirectly lead to the occurrence of DR, resulting in the appearance of Microaneurysm, Fibrotic membrane, and New blood vessels and so on in the retina. These factors are interrelated and thus contribute to the development of DR.

**FIGURE 3:**
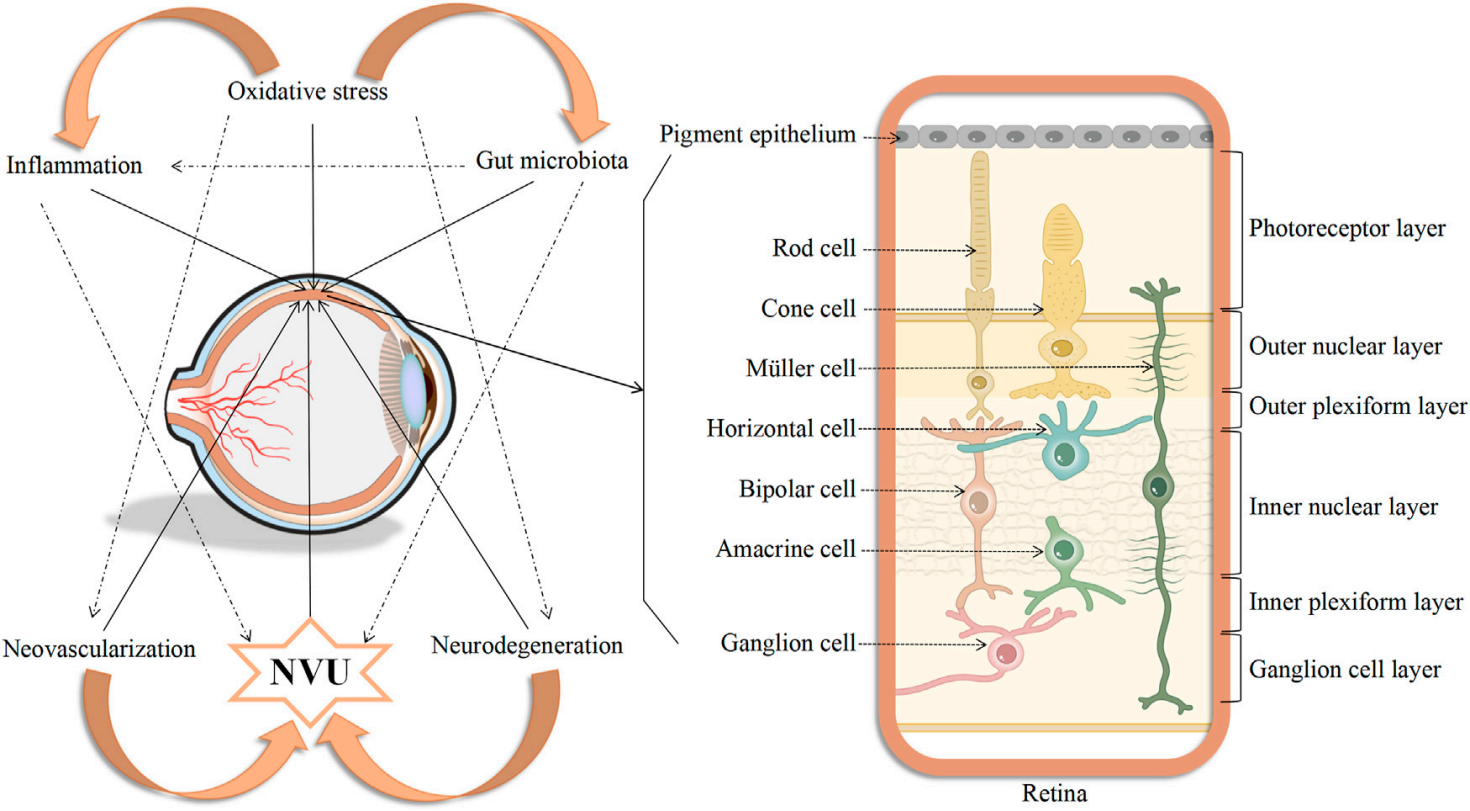
Diagram showing the interconnectedness between oxidative stress, inflammation, neovascularization, neurodegeneration, neurovascular unit, and gut microbiota, as well as the simulated picture of cells affected in the retina of DR.

In summary, the pathophysiological mechanisms underlying DR are complex and include a variety of contributory factors, such as oxidative stress, inflammation, neovascularization, neurodegeneration, the NVU, and the gut microbiota. Oxidative stress is one of the primary triggers of inflammatory cascades and, in addition, both oxidative stress and inflammation increase the levels of retinal autophagy, leading to cellular dysfunction, necrosis, apoptosis, and death. The affected cells may be nerve cells, endothelial cells, pericytes, RPE cells, and glial cells which, in turn, lead to further abnormalities in neovascularization, neurodegeneration, and the NVU. All these factors may have subtle interactions with each other, ultimately contributing to the development of DR (Figure 2). However, more evidence is required on the factors controlling DR progression. Therefore, the integration of knowledge on these mechanisms allowing the construction of a complete network of factors contributing to the disease will surely provide sufficient theoretical basis for formulating new treatment strategies based on current clinical diagnosis and treatment (Figure 3).

Although the etiology and pathology of DR has been extensively studied for half a century, its treatment still presents great challenges. Conventional drugs can be used to control hyperglycemia, hypertension, and hyperlipidemia. However, the effects of these basic treatment methods are limited. There are also some targeted treatments for DR, such as topical surgery, topical laser therapy, and single-component drugs (anti-VEGF or steroid drugs). However, surgical treatment is only indicated for PDR cases with non-clearing vitreous hemorrhage or traction retinal detachment. Some new methods are replacing traditional laser treatment methods, including pattern scanning laser (PASCAL), micropulse technology, and the navigation laser system (NAVILAS). Although anti-VEGF drugs are the first-line treatment strategy, there are still many limitations associated with the use of a single treatment strategy. It is possible that as more therapeutic strategies and critical molecules are discovered that anti-VEGF drugs may cease to be first-line drugs for the treatment of DR and VEGF may be supplanted as the star molecule of DR. As mentioned above, there are several factors that have great potential to be new targets for DR therapy, including SCGIII, miRNAs, and the gut microbiota. In addition, new therapeutic drugs are also being developed, such as cardiolipin targeting peptide (MTP-131), lipoic acid, lutein, ARA290, and dalapradib (Wang and Lo, 2018). Unfortunately, there are currently no therapeutic strategies that can fully reverse the retinal damage caused by DR. Proposed new treatment strategies include traditional Chinese medicine treatment, targeted therapy, gene therapy, stem cells therapy, and nanotechnology. Although most of these have not yet entered clinical trials, it is likely that there will be earlier and faster detection of the pathophysiological changes in DR in the future. We hope to observe the progression of a patient’s disease in a more individualized and longitudinal manner to avoid the occurrence and development of DR.
